# In situ AFM visualization of Li–O_2_ battery discharge products during redox cycling in an atmospherically controlled sample cell

**DOI:** 10.3762/bjnano.10.94

**Published:** 2019-04-24

**Authors:** Kumar Virwani, Younes Ansari, Khanh Nguyen, Francisco José Alía Moreno-Ortiz, Jangwoo Kim, Maxwell J Giammona, Ho-Cheol Kim, Young-Hye La

**Affiliations:** 1 IBM Almaden Research Center, 650 Harry Road, San Jose, CA 95120, USA; 2 Repsol Technology Center, Agustin de Betancournt s/n, 28935 Motstoles, Madrid, Spain

**Keywords:** AFM, battery, EIS, in situ, Li–O_2_

## Abstract

The in situ observation of electrochemical reactions is challenging due to a constantly changing electrode surface under highly sensitive conditions. This study reports the development of an in situ atomic force microscopy (AFM) technique for electrochemical systems, including the design, fabrication, and successful performance of a sealed AFM cell operating in a controlled atmosphere. Documentation of reversible physical processes on the cathode surface was performed on the example of a highly reactive lithium–oxygen battery system at different water concentrations in the solvent. The AFM data collected during the discharge–recharge cycles correlated well with the simultaneously recorded electrochemical data. We were able to capture the formation of discharge products from correlated electrical and topographical channels and measure the impact of the presence of water. The cell design permitted acquisition of electrochemical impedance spectroscopy, contributing information about electrical double layers under the system’s controlled environment. This characterization method can be applied to a wide range of reactive surfaces undergoing transformations under carefully controlled conditions.

## Introduction

Italian anatomist Luigi Galvani [1] is credited with the birth of electrochemistry in the year 1791. Electrochemistry is the study of chemical processes that cause electrons to move from one element to another causing oxidation (loss of electrons) and reduction (gain of electrons) reactions. Hence electrochemical phenomena form the basis of battery technologies that provide power to modern day mobile electronics. Being inherently atomic/molecular in origin, there has been significant interest in understanding electrochemical phenomena of these redox materials at the micrometer and nanometer scales. Gewirth et al. [2] reviewed the use of scanning tunneling microscopy (STM) and atomic force microscopy (AFM) investigations of phenomena such as reconstructions, restructuring and adsorption of ions. Phenomena such as under-potential deposition [3], corrosion and molecular adsorbates on a variety of surfaces [4] have also been investigated with scanning probe microscopy. In situ local probe techniques at electrical interfaces [5] use scanning probe microscopy to probe surface changes and reactions. A recent review by Yang et al. [6] discusses various in situ techniques to monitor electrochemistry of rechargeable battery materials. When considering rechargeable battery materials, electrochemical reactions [7] between lithium (Li) and oxygen (O_2_) offer the highest theoretical potential of any possible battery technology of 3500 Wh/kg. Li and O_2_ reactions have been the subject of intense experimental and theoretical investigations [8–17]. Of particular relevance are investigations that shed light on the morphological changes that occur on the electrodes during the Li/O_2_ electrochemical reactions. Jung et al. [18] used transmission electron microscopy to investigate electrochemical processes of Li/O_2_ cells. In situ observations using electron beams tend to have limited time for observation as the electron beam reacts with the Li and the Li/O_2_ discharge products. Lu et al. [19] used ambient pressure X-ray photoelectron spectroscopy to study the reactions under ultrahigh vacuum as well as 500 mtorr O_2_ pressure. Zheng et al. [20] performed in situ scanning electron microscopy of the reaction whereby they observed the growth of toroidal lithium peroxide (Li_2_O_2_) particles along a specific direction as opposed to a single point. Yu et al. [21] performed in situ UV–vis absorption spectroscopy, surface enhanced Raman vibrational spectroscopy and ex situ infrared spectroscopy of O_2_ reduction and evolution reactions respectively. Lim et al. [22] used X-ray diffraction to study surface changes resulting from Li/O_2_ reactions. Wen et al. [23] performed in situ AFM imaging of the Li/O_2_ electrochemical reaction on highly oriented pyrolytic graphite (HOPG). In their work, the imaging during the electrochemical reaction was performed using contact mode and upon completion of the electrochemical reaction, the surface was cleaned and then imaged using tapping mode. Liu et al. [24] performed in situ AFM investigations of Li/O_2_ electrochemistry measuring formation of toroidal and spherical structures. The AFM scanner was briefly exposed to the atmosphere in their case, leading to possible increases in the amount of water in the electrolyte. In our previous attempt [25] a closed AFM cell was exposed to atmosphere during imaging and discharge with oxygen saturated solvent precluding any impedance spectroscopy and cell recharge studies. Lang et al. discussed in situ AFM studies of lithium/sulfur [26] batteries. Thus rigorous environmental control and time domain correlation of discharge products to electrochemical voltages remain as the challenges for highly reactive Li/O_2_ and other electrochemical systems.

In this work, we present in situ morphological investigations of Li/O_2_ electrochemistry products using tapping mode AFM with complete time domain correlated visualizations recorded during discharge and recharge cycling. The voltage and capacity of an electrochemical Li/O_2_ cell were simultaneously monitored and correlated with the evolution of nano- and micro-structured discharge products. In contrast to many of the previous studies mentioned above, improvements in cell design have allowed us to keep the battery cell completely sealed during discharge, charge, and collection of electrochemical impedance spectra. The controlled atmosphere allowed our study to trace the topographical changes on the cathode while minimizing the chance of exposure to external sources of contaminants. The electrolyte consisted of lithium nitrate (LiNO_3_) as a salt in tetraethylene glycol dimethyl ether (TEGDME) solvent containing three concentrations of water: <20 ppm, ≈2500 ppm and ≈4600 ppm. Water has been added in the electrolyte in multiple previous studies [27–29] to increase cell capacity at elevated concentrations, suggesting the possible catalytic role in Li/O_2_ reactions. However, without the stringent environmental controls, as presented in our study, the electrolyte could lose water over time to the surrounding moisture and oxygen-free glove box, leading to erroneous interpretations of the data. By minimizing the water loss, our study allows for stable analysis of the vivid changes of the cathode surface for short (≈hours) and relatively long time (≈days) experiments.

## Results and Discussion

### In situ AFM cell design

**Figure 1:**
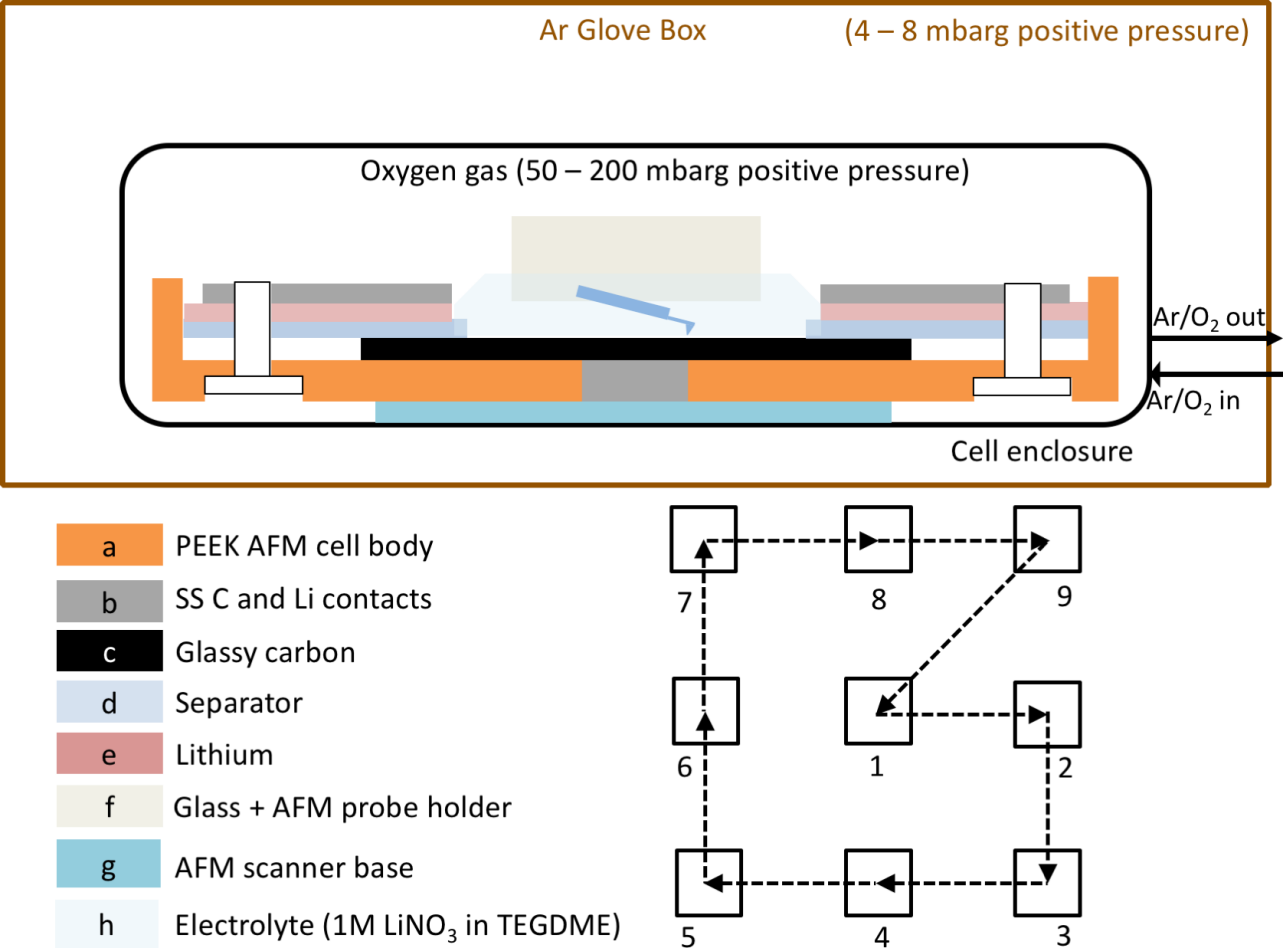
Schematic drawing of the AFM cell. Specially designed flanges on the sides of the glove box were used to accommodate all-metal gas feeds and electrical connections to the potentiostat and AFM controller. A spiral pattern (1–9) was devised to minimize the impact of tapping mode AFM scanning on the electrochemical deposits. This allowed every AFM line scan to be correlated with the electrochemical changes on the glassy carbon cathode surface.

The Li/O_2_ cell consisted of a polyetheretherketone (PEEK) cell body (Figure 1a) with a stainless steel (SS) stub (Figure 1b) that allowed electrical contact to glassy carbon cathode (Figure 1c) as shown in Figure 1. For AFM experiments, the design challenge was to allow the AFM tip contact with the glassy carbon surface while simultaneously allowing the electrochemical reaction between Li^+^ and O_2_ to occur at the solvent–cathode interface. This was necessary to keep the glassy carbon surface available for the AFM tip, the lithium ions and oxygen at the same time. This necessitated the use of a donut-shaped polyethylene separator (Figure 1d) and lithium (Figure 1e) anode. The electrical contact to the lithium anode was made using stainless steel (Figure 1b). The entire assembly was performed inside of the glove box (<0.1 ppm H_2_O and O_2_), allowing the assembled cell to be free of moisture and oxygen exposure. The cell components were held together using Nylon screws outside of the glassy carbon discs, preventing any contact between screws and the electrolyte. It should be noted that the cell design was optimized to allow for the in situ study of the Li/O_2_ electrochemical reaction rather than for the highest capacity. The main aim was to obtain electrochemical impedance spectroscopy (EIS), discharge/recharge voltages and capacities time-domain-correlated with AFM images of topography, all in a completely atmospherically isolated and controlled setting. In our recent study [30] using this in situ AFM set-up we monitored surface changes on the products of a Na–oxygen discharge reaction. The terms electrochemical cell and battery are used interchangeably in this study.

Upon cell assembly, electrical connections were made between the lithium and carbon stainless steel contacts and the potentiostat inputs. The electrical resistance between them was <10 ohms. This low resistance allowed us to perform EIS. The cell was placed in a glass enclosure on the AFM. A leak-free ultrapure oxygen line from a custom designed glove box flange was connected to the cell enclosure of the AFM. The electrolyte chosen was 1 M LiNO_3_ in TEGDME with <20 ppm, ≈2500 ppm and ≈4600 ppm of H_2_O. Previous studies [12,31–32] have reported formation of varied micro and nanostructured discharge products as a function of water concentration using the same salt. 80 μL of electrolyte was used in the electrochemical cell, which was placed on the AFM scanner base (Figure 1g). The enclosure was then sealed with the probe holder (Figure 1f). The oxygen pressure in the cell was maintained at 100–200 mbarg [33]. O_2_ sensors in the glove box always registered an O_2_ concentration in the glove box of <0.1 ppm throughout the experiments. Prior to lowering the probe into the fluid electrolyte, EIS was performed on the cell using a BioLogic VMP3 potentiostat at frequencies between 1 MHz and 100 mHz with 7 points per decade and an alternating current (AC) amplitude of 3 μA. The probe holder was then lowered into the electrolyte and a second EIS scan was performed after two hours of solvent oxygenation. This is in contrast with previous such studies [23–24] where a pre-oxygenated electrolyte was used. The cell design was such that the AFM probe landed in the middle of the 1 mm^2^ area of the glassy carbon disc for all experiments. The AFM probe oscillated with a 10 nm peak-to-peak amplitude away from the surface while submerged in electrolyte. The surface engaged amplitude of the probe was 7.5 nm peak-to-peak. The scan rate was 2 Hz for a 3 μm scan size. Since tapping mode scanning could have a propensity to change the surface morphology over long scanning periods, we employed a spiral image collection scheme, which was designed to minimize the effect of scanning on the structures generated during the Li/O_2_ electrochemistry (Figure 1). The spiral scheme was composed of nine images of 3 × 3 μm each, collected from center to periphery. The distance between the 3 μm scans was 8 μm center-to-center. This allowed the sampling of multiple areas on the glassy carbon surface while ensuring that the impact of AFM scanning on the deposits was minimized. In addition, the spiral scan scheme returned to the original scan area every 10th scan thus allowing correlation of time-domain information with cell voltage and capacity.

### Electrochemical impedance spectroscopy (EIS)

**Figure 2:**
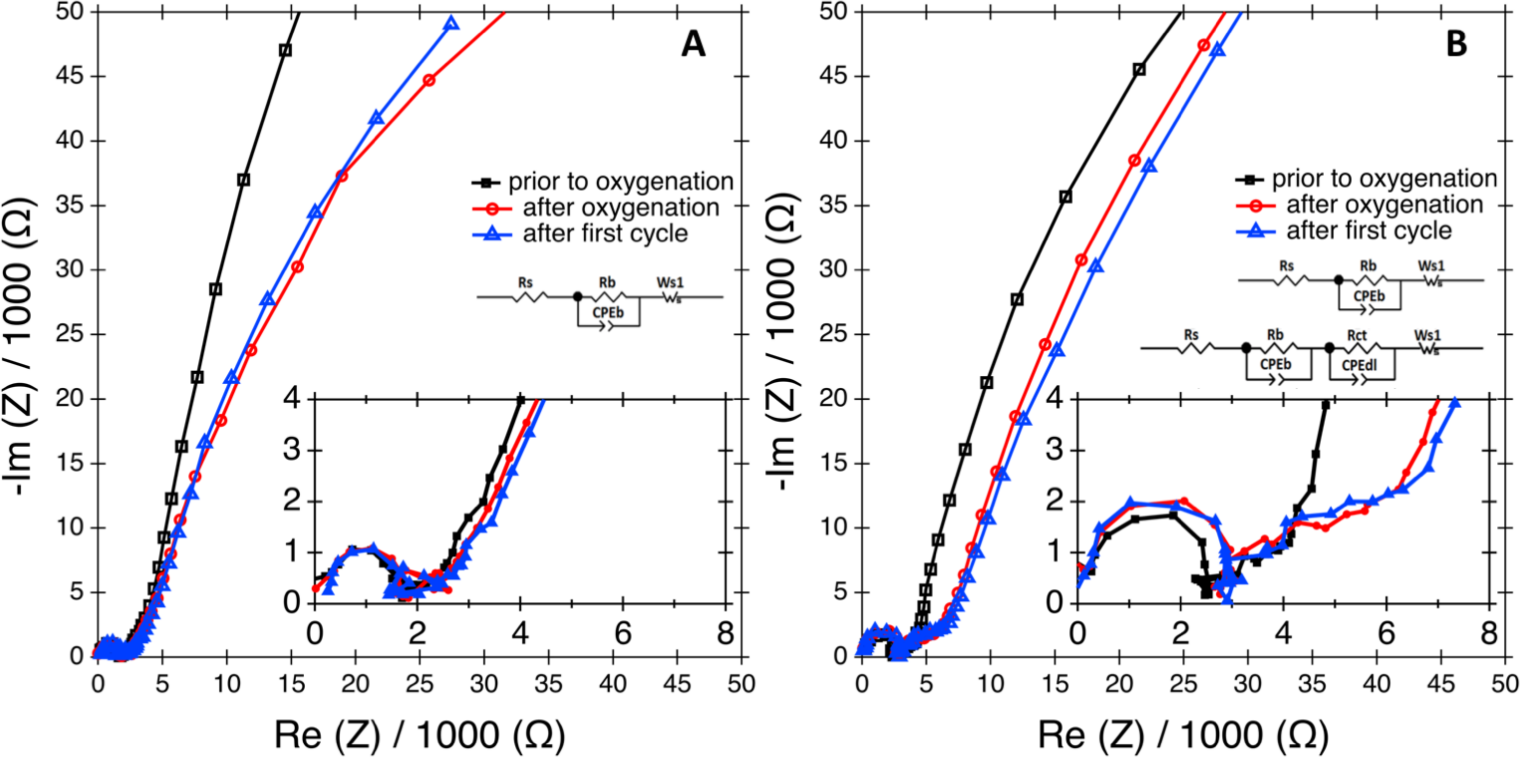
Electrochemical impedance spectroscopy (EIS) curves collected before (square points) solvent oxygenation, after solvent oxygenation for two hours (circle points) and after the first discharge–recharge cycle (triangle points). The EIS data were collected for cells with 1 M LiNO_3_ in TEGDME with <20 ppm (A) and 2500 ppm (B) of water in the electrolyte. Fitting parameters used in the equilvent circuit mode(s) shown in the inset are presented in Table S1 of Supporting Information File 1. Prior to oxygenation, and for all EIS measurements with <20 ppm water, an equivalent circuit with a constant phase element (CPEb) was used and the rest of the EIS spectra were fit with equivalent circuits that included both CPEb and CPEdl.

EIS [34] was employed to measure the impedance of the AFM electrochemical system over the range of frequencies between 1 MHz and 100 mHz. This frequency response of the system was used to characterize the cell impedance at different stages of cycling [35]. The unique cell, enclosure and glove box design permitted the study of the changes in EIS properties [36] of the electrochemical system before and after oxygenation of the electrolyte in contrast to prior AFM studies [23–24] that have used oxygen saturated solvents. Figure 2 shows Nyquist plots prior to oxygenation, after oxygenation and after the first discharge/recharge process for Li–O_2_ batteries prepared from electrolytes containing <20 ppm (A) and ≈2500 ppm (B). The plot for ≈4600 ppm water is presented in Figure S1 of Suppoting Information File 1. The insets show the magnified high-frequency region and the equivalent circuit model for the cell prior to oxygenation (top), after oxygenation and the first cycle (bottom). For <20 ppm of water only one equivalent circuit model was used.

All Li–O_2_ cells prior to oxygenation demonstrate a high frequency semi-circle, while after introducing the oxygen to the 2500 ppm water and 4600 ppm water cells, a mid-frequency semi-circle appears that persists during the subsequent discharge/recharge cycle, as presented in the high-magnification Nyquist plot inset of Figure 2. This mid-frequency circle is notably absent in the case of the cell with <20 ppm of water. To construct an equivalent circuit model, the resistance between the electrode and current collectors is represented by Rs. The passivated film on the electrode has an interfacial resitance given by Rb. Addtionally, the electric representation of the passivated film contains a constant phase element, CPEb. The charge transfer resistance upon oxygenation and cycling results in a semicircle appearing in the mid-frequency region. Rct denotes this charge transfer resitance and is related to the kinetics of the reaction taking place at the cathode [37]. The constant phase element CPEdl represents the capacitance of the double layer formed at the cathode. The diffusion of oxygen and lithium ions in the electrolye results in a low-frequency linear Warburg component, Ws. The slope of the Warburg impedance declines after introducing oxygen, indicating an increase in the migration resistance, while the diameter of the semicircle remains almost unchanged after oxygenation, suggesting a stable interfacial resistance in the cell. The increasing impedance right after the Warburg region observed in the sample after oxygenation [37] indicates surface and pore blockage on the glassy carbon cathode caused by diffusion of oxygen into the carbon. This could be attributed to the absence of a separating layer between the glassy carbon and the liquid electrolyte [38]. The fitting parameters for the equivlent circuit model are given in Table S1 in the Supporting Information File 1. Thus this study enables direct EIS investigations of electrochemical systems in a controlled environment and clearly documents differences in the spectra due to the presence of water in the solvent that, by modifying the charge transfer resistance, improves the kinetics of the reaction.

### Topographical observations using AFM

**Figure 3:**
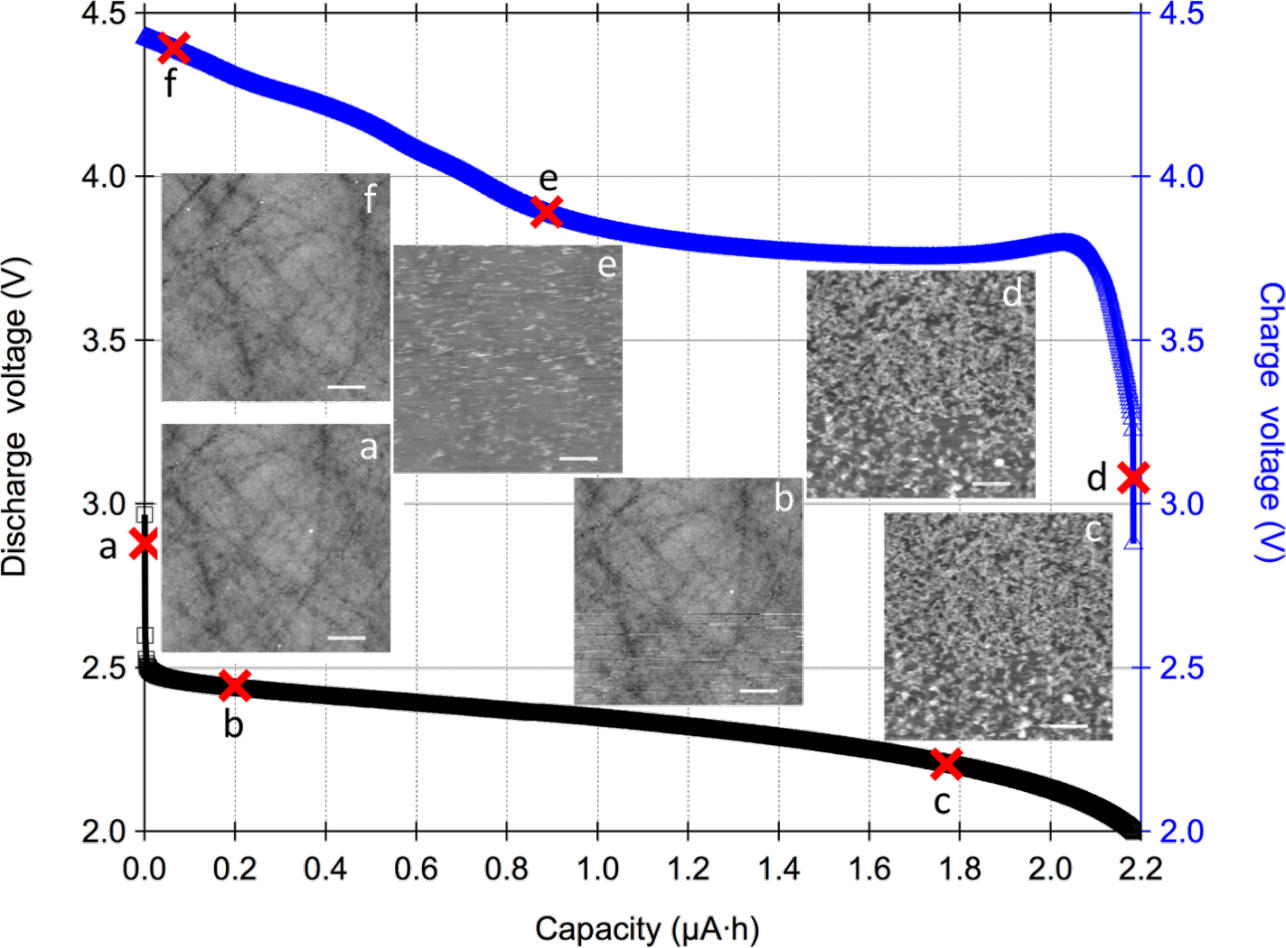
Electrochemical discharge (black, left *y*-axis) and recharge (blue, right *y*-axis) curves with topography images from simultaneous AFM scans. The X marks on the curves denote the discharge or recharge capacity at the end of the AFM scan. The AFM images (3 × 3 µm) were collected in 1 M LiNO_3_ in TEGDME with <20 ppm of water in the electrolyte from one of the nine spiral spots as shown in Figure 1. The discharge current was 5 µA. Electrochemical deposits appear to nucleate, grow and then eventually shrink on the glassy carbon surface as the cell goes through discharge and recharge. The Z scale for Figure 3a, 3b, 3e and 3f is 20 nm and 80 nm for Figure 3c and 3d; the horizontal scale bar is 500 nm. 63 images in total are collectively presented in Movie 1 (Supporting Information File 2) for time domain visualizations of all the nine areas scanned on the surface.

**Figure 4:**
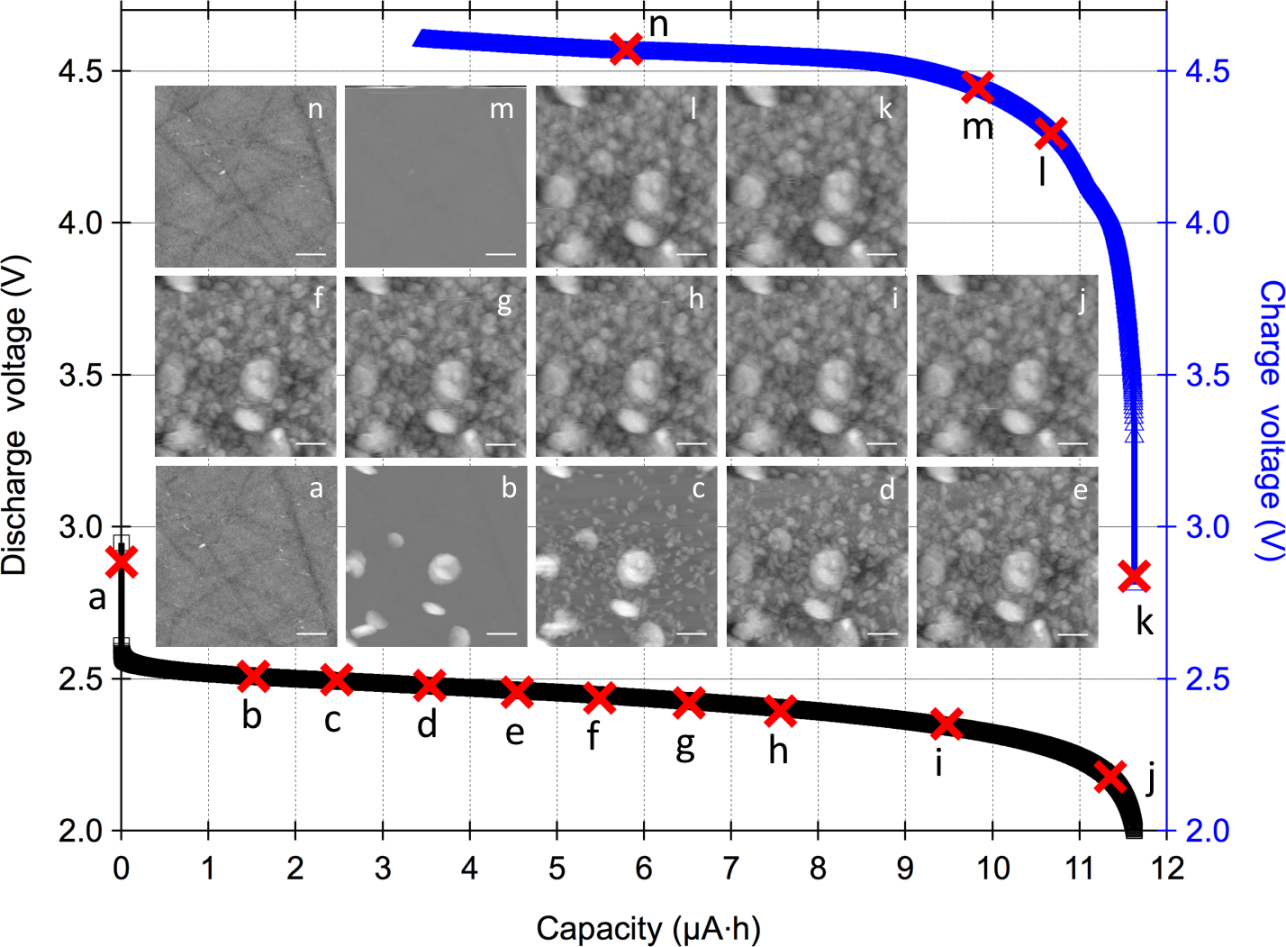
Electrochemical discharge (black, left *y*-axis) and recharge (blue, right *y*-axis) curves with topography images from simultaneous AFM scans. The X marks on the curves denote the discharge or recharge capacity at the end of the AFM scan. The AFM images (3 × 3 µm) were collected in 1 M LiNO_3_ in TEGDME with ≈2500 ppm of water in the electrolyte from one of the nine spiral spots as shown in Figure 1. The discharge current was 5 µA. Nanostructured electrochemical deposits abruptly appear (≈4% capacity) on the cathode surface during the initial stages of discharge. Figure 4b is at 12.7% discharge capacity. In Figure 4c–e smaller nanostructures appear to nucleate and grow on the cathode surface. In Figure 4f–j the size of the deposits continues to increase along with fresh nucleation and growth on all areas. During recharge the nanostructures abruptly disappear between 14% and 18% of the recharge capacity with the last of the deposits captured in three traces in Figure 4m. The Z scale for Figure 4a and 4n is 50 nm and Figure 4b–l is 500 nm. 184 total images are collectively presented in Movie 2 (Supporting Information File 3) for time domain visualizations of all the nine areas scanned on the surface.

AFM was used to monitor morphological changes on the glassy carbon cathode surface. The topography images in Figure 3 and Figure 4 each show one of the nine scanned regions at two different water concentrations studied with the corresponding discharge (left, black color) and recharge (right, blue color) curves from the first cycle. In Figure 3 the electrolyte contained <20 ppm of water, and in Figure 4 the electrolyte contained ≈2500 ppm of water. The main aim was to study the resultant increase in the cell discharge capacity at increased water concentration and the corresponding morphological changes on the glassy carbon cathode with this technique. For all measurements, the discharge was cut-off at 2 V vs Li and the recharge was cut-off at 4.6 V vs Li or 100% state-of-charge (SOC). Across different water concentrations the discharge and recharge currents were 5 µA.

Figure 3 shows AFM topography images scanned on the glassy carbon surface with electrolyte containing <20 ppm of H_2_O. Prior to the start of the discharge reaction in Figure 3a at an open-circuit potential of 2.921 V, the surface of the glassy carbon is smooth and free of deposits. At about 5% discharge capacity, deposits nucleate on the surface of the glassy carbon in the first scan in Figure 3b (and Movie 1, Supporting Information File 2). In Figure 3c, the cell has reached about 80% of its discharge capacity and nanostructured deposits cover most of the glassy carbon surface. The deposits have rod-like, partial spherical and spherical shapes. The measured deposits are 200–400 nm at 100% discharge capacity of 2.2 µAh. The deposits do not appear to adhere preferentially to surface features on glassy carbon such as polishing marks. During the initial stages of recharge in Figure 3d the surface state is similar to that at about 80% discharge capacity. Upon recharging the cell to about 60% SOC the surface of glassy carbon starts to reappear in Figure 3e as the deposits shrink in size. In Figure 3f at >98% SOC the glassy carbon appears to be topographically restored to its original state with no evidence of residual deposits although chemically the surface might be modified. AFM topography images and the corresponding cell capacity established a baseline for our technique. As expected, with <20 ppm of water in the electrolyte, the cell had a relatively small discharge capacity of 2.2 µAh. Despite reaching the prescribed voltage cut-off, it is clear from the AFM images that the capacity of this cell is not limited by electrode surface area (a failure mechanism known as electrode cloging) as free areas of the electrode surface are still visible. We speculate that under these conditions the cell dies due to the depletion of O_2_ as a result of slow transport through the electrolyte limited by diffusion in contrast with conventional differential electrochemical mass spectrometry (DEMS) cells where oxygen is bubbled through the electrolyte.

Figure 4 shows AFM topography images from one of the nine regions (Movie 2, Supporting Information File 3 for all the nine regions) scanned on the glassy carbon surface for electrolyte containing ≈2500 ppm of H_2_O. In Figure 4a, prior to the start of the discharge reaction at an open-circuit potential of 2.948 V, the surface of glassy carbon is smooth and free of electrochemical deposits. At about 4.3% of discharge capacity, the first nanostructures suddenly appear on the glassy carbon surface. In Figure 4b, the cell is at 12.7% discharge capacity and displays partial and complete toroidal structures on the glassy carbon surface. The initial deposits on the carbon surface are the largest (≈400 nm) compared with the products formed during the rest of the discharge reaction. Similar to the case in Figure 3c, the deposits do not form at preferential sites on the glassy carbon. In Figure 4c, at 21.6% discharge capacity, multiple partial and complete toroidal structures on the surface are a fraction of the size of the original toroidal structures. In Figure 4d–j, when the cell discharges to 92.3% of its capacity, the structures that appeared as deposits from the solution as well as those formed due to nucleation on the surface of glassy carbon grow in volume while fresh nucleation and growth of toroidal structures continues on all areas. The total discharge capacity of the cell was measured to be 11.6 µAh. Upon completion of discharge, the cell was allowed to rest at open-circuit voltage for 10 min prior to the recharge process. In Figure 4l, the structures formed on the surface persist until the cell has reached about 14% of SOC. Recharge beyond that SOC (Figure 4m) results in an abrupt disappearance of deposits from the surface into the surrounding electrolyte solution. Between 14% and 18% SOC, all the deposits disappear from the glassy carbon surface. Figure 4n shows that the surface of glassy carbon continues to be free of deposits for the rest of the recharge process. While the static images presented in the main paper convey information about a single 3 × 3 µm area scanned on the glassy carbon surface, vivid information about the dynamics of deposit formation during discharge and dissolution during recharge is visualized in Movie 2 (Supporting Information File 3). Each of the nine areas scanned in a spiral pattern shows the similar behavior of the sudden appearance of electrochemical deposits from solution and nucleation and growth of smaller deposits on the surface of the glassy carbon during discharge. Complementarily, the sudden disappearance of the electrochemical deposits during recharge occurs in a small voltage window of 4.25 V (14% SOC) to 4.35 V (18% SOC). As expected, compared to the baseline presented in Figure 3, the cell capacity increased by a factor of 5.3 as the amount of water increased from <20 ppm to ≈2500 ppm. Simultaneously acquired AFM data reveals a stark contrast wherein large electrochemical deposits appear suddenly on the glassy carbon surface in the study with electrolyte containing ≈2500 ppm of water.

**Figure 5:**
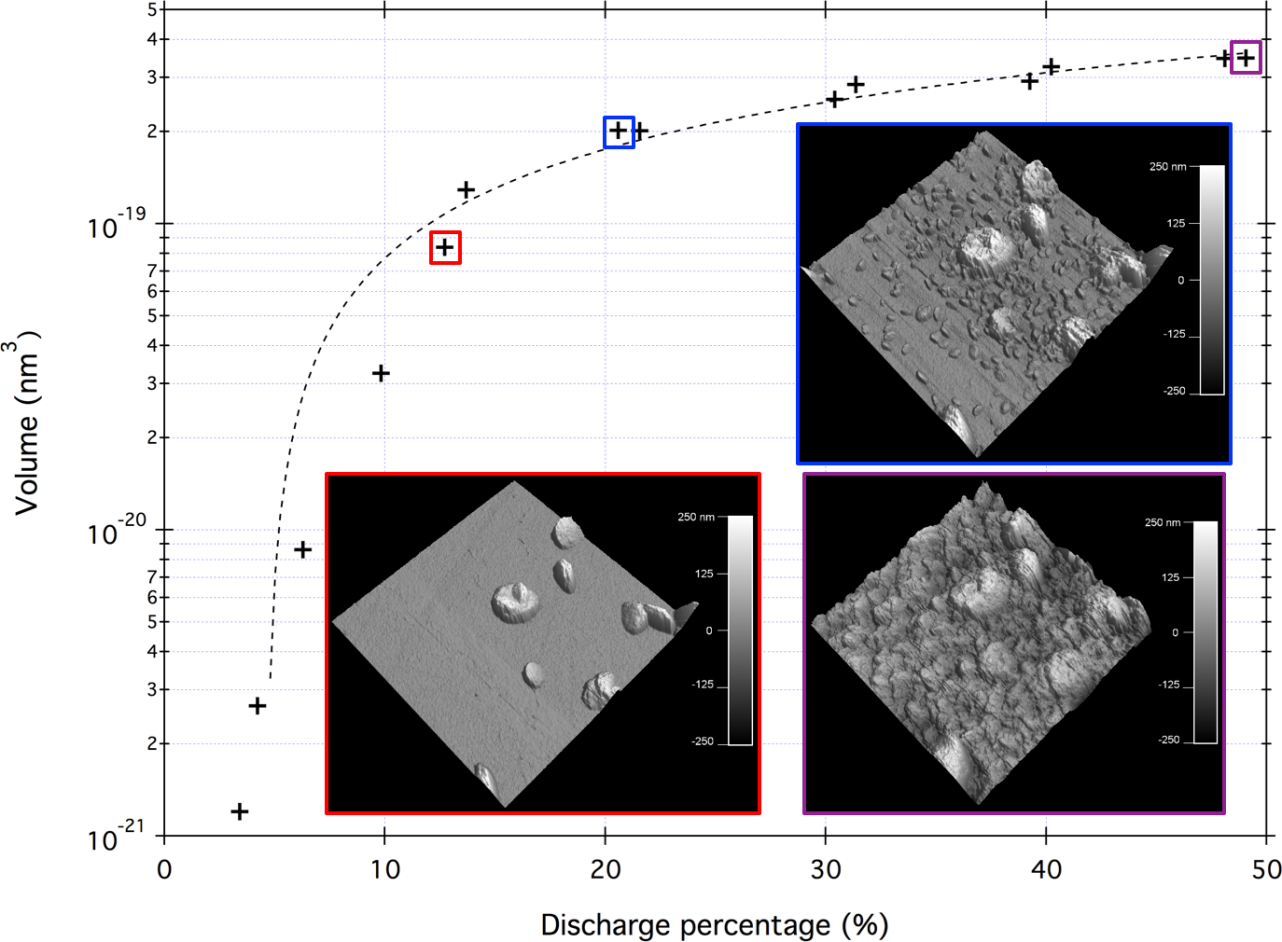
3D volume analysis of electrochemical deposits formed on the glassy carbon surface in Figure 4. The red inset shows a 3D view of the surface at ≈12% discharge capacity. The nanostructures deposited on the surface exhibit platelet morphology. An even finer nanostructure was observed beyond the platelet morphology. The blue inset shows a 3D view of the surface at ≈22% discharge capacity. In addition to the nanostructures deposited on the surface, much smaller hemispherical nanostructures appear to nucleate and further grow. The purple inset shows the morphology at 100% discharge.

This in situ technique allows a close approximation of the three-dimensional volume of the electrochemical deposits formed during discharge. Figure 5 shows the measured deposit volume as a function of the cell discharge capacity. The graph reveals that during the initial stages, no discharge products appear in the images. At a discharge capacity of ≈4% the first deposits appear on the surface beyond which the measured volume of the precipitates can be fit to a second-order polynomial function of the cell discharge capacity. At cell discharge capacities beyond 50% a good estimate of the volume cannot be obtained from the measured topography because the electrochemical deposits obscure the reference plane of the glassy carbon surface. The insets in Figure 5 show the three-dimensional structure of the toroids from Figure 4 at different discharge capacities. These images reveal that the toroids have a fine nanostructure, as measured previously [39]. The end radius of the AFM tip, estimated to be between 5 nm and 10 nm, limits the image resolution.

Motivated by our measurements of increased capacity at ≈2500 ppm water, the water concentration was further increased to ≈4600 ppm. A similar sudden appearance of electrochemical deposits on the surface followed by further nucleation and growth during discharge and disappearance during recharge was also measured with a corresponding increase in the cell capacity to about 24 µAh (Movie 3, Supporting Information File 4). This represents an ≈11-fold increase in the capacity. However, at 4600 ppm of water in the electrolyte, the AFM probe chosen for this study is fouled by the nanostructures and unable to cleanly image, as evidenced in Movie 3 (Supporting Information File 4). At high water concentrations (>1 vol %) in the solvent, the lithium metal tends to react vigorously with the water, thus such processes cannot be studied using this technique. Additionally, when the size of the discharge products exceeds 1/4 of the Z-range of the piezo (4 μm in our case) the technique cannot be used to effectively monitor the reaction.

This electrochemical study of the Li/O_2_ reaction in a highly controlled environment clearly documents the role that water plays in increasing the cell capacity from 2.2 µAh with <20 ppm water to 24 µAh with ≈4600 ppm, establishing the validity of the technique. The corresponding visualization of electrochemical discharge products on the surface of glassy carbon during the Li/O_2_ reaction reveals that when the electrolyte contains ≈2500 ppm and ≈4600 ppm water, nanostructures of the size of about 500 nm suddenly appear during the initial phases of the discharge reaction (less than 10% of the discharge capacity). The size of these initial nanostructures increases as the amount of water in the electrolyte increases. The growth on the surface that follows during discharge consists of smaller nanostructures (<200 nm) that then grow to the larger sizes and eventually the entire surface is covered by the growths. During this time, the original nanostructures also continue to grow. At the cell cut-off voltage, the entire surface is covered by micro- and nanostructures for the electrolytes containing ≈2500 ppm and ≈4600 ppm of water. In contrast, when the electrolyte contains <20 ppm water, the surface is not fully covered by nanostructure growth. This is reflected in lower recharge voltages for the electrolyte containing <20 ppm of water. Thus, our initial AFM observations support the hypothesis that during cell discharge, the presence of water increases superoxide ion solubility and diffusivity in the electrolyte, resulting in at least a part of the reaction occurring in the electrolyte, as suggested by previous studies [12,39–40]. Similarly, during recharge the deposits abruptly disappear from the surface at less than 50% SOC, likely remaining suspended (or dissolved) in the solution. Thus, even though solution-mediated processes have been proposed for discharge, this study suggests that such processes also influence cell recharge. The main take away message is the ability of this technique to shed light on such processes in conjunction with other complimentary techniques.

**Figure 6:**
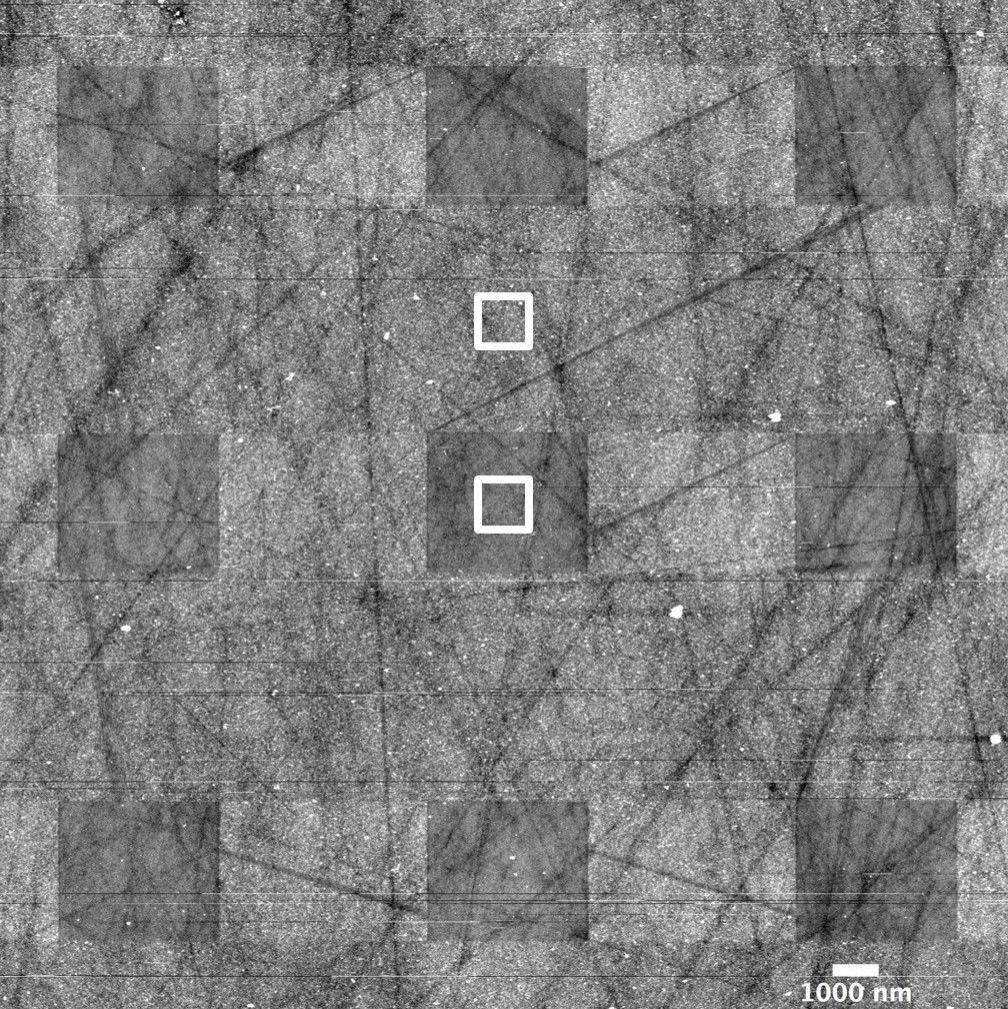
22 µm scan of the glassy carbon surface collected at the end of electrochemical discharge in 1 M LiNO_3_ in TEGDME with ≈4600 ppm of water in the electrolyte at discharge current of 350 nA. The experiment lasted for 7 days with the AFM probe continuously scanning the surface in a spiral pattern. More than 3000 tapping mode images were acquired during this time. The average difference in the root mean square Z-values in the two square boxes was 1.5 nm. Thus 344 tapping mode scans change the height of electrochemical deposits on the surface only by a total of only 1.5 nm! Movie 4 (Supporting Information File 5) shows a subset of more than 3000 images along with the discharge curve, documenting subtle changes on the glassy carbon surface at slow discharge currents.

We have quantified the influence of scanning in tapping mode on electrochemical deposits on the electrode surface during cell discharge. A slow discharge at 350 nA current was performed using ≈4600 ppm water containing electrolyte to scan the carbon surface for 7 days in a spiral pattern (Figure 6). This resulted in >3000 images being acquired with the same probe (due to file size limitations a subset of images is presented in Movie 4, Supporting Information File 5). A larger scan performed at the end allowed us to compare differences in height between the area scanned once and the area scanned 344 times. The two rectangles in Figure 6 show an average Z-height difference in the two areas of about 1.5 nm after 344 scans, suggesting minimal impact on the surface topography of tapping mode scanning during electrochemistry! This also indicates that even relatively slow electrochemical reactions can be effectively monitored using this technique. The electrochemical deposits at 350 nA discharge current grew conformal to the surface of glassy carbon, which is in contrast with a previous report [41]. The origins of such conformal growth are under further investigation.

## Conclusion

A sealed AFM cell permitting in situ scanning probe microscopy observation of electrochemical processes was designed, fabricated, and operated within the controlled atmosphere of a glove box. An example Li/O_2_ battery system in 1 M LiNO_3_ in TEGDME was studied at three different water concentrations. The electrochemical impedance spectra collected from the AFM cell allowed for the study of cell impedance before and after cycling in the Li/O_2_ battery. Time-domain correlated images were collected showing changes in surface topography while cell discharge and recharge voltages/capacities were measured. The reversible formation of reaction products was observed in the process of initial precipitation from solution, followed by surface nucleation and growth during discharge, and abrupt disappearance of deposits during recharge. An 11-fold increase in the cell discharge capacity was measured as the water concentration in the electrolyte increased from <20 ppm to ≈4600 ppm. The imaging protocol was designed to minimize the impact of the AFM technique itself on the measured results. This in situ AFM study highlights the potential of this technique in elucidating other electrochemical systems where stringent environmental control is critical for desired outcomes. Our results indicate that for the Li/O_2_ system, some electrochemical processes may occur in the solution, especially for electrolytes containing ≈2600 ppm and ≈4600 ppm of water. In situ AFM, however, is not a tool for characterizing species in solution and complementary analysis techniques should be used to further understand the mechanism of the reaction in a similarly controlled environment. The authors hypothesize that an ability to track changes on the cathode surface during electrochemical reaction of highly reactive species (such as lithium and oxygen) in a controlled environment proves a need for such complimentary analyses, as understanding redox interactions is inherently complex.

## Experimental

A Cypher atomic force microscope (AFM) with an environmental scanner from Oxford Instruments operating inside of an mBraun glove box was used in this study. AC 160TS AFM probes from Olympus were used for all the experiments and were secured to the probe holder with a polyetheretherketone (PEEK) clip. The glassy carbon for the AFM experiments was obtained from Tokai carbon products and was certified to have alkali metal impurities less than 1 ppm [42]. Glassy carbon was laser cut into 12 mm diameter discs. The surface of the glassy carbon was polished [43] to an average root-mean-square roughness of less than 10 nm. Upon polishing, the glassy carbon was cleaned with isopropyl alcohol and deionized water and further dried in a vacuum oven at 120 °C for at least 12 h. The glassy carbon was transferred to the glove box whilst hot from the vacuum oven to minimize any chance of moisture adsorption on the carbon surface. The TEGDME solvent was dried in molecular sieves for multiple days in a glove box before being used for AFM experiments. The cell components were thoroughly cleaned and dried before each experiment.

## Supporting Information

File 1 Additional figure, table and movie captions.

File 2 Movie 1.

File 3 Movie 2.

File 4 Movie 3.

File 5 Movie 4.
