# Supplementary material for: In situ AFM visualization of Li–O2 battery discharge products during redox cycling in an atmospherically controlled sample cell
Source: Beilstein J Nanotechnol. 2019 Apr 24;10:930–40. doi: 10.3762/bjnano.10.94 (PMC6541370; doi:10.3762/bjnano.10.94)
Supplement: File 1 — Additional figure, table and movie captions. [file Beilstein_J_Nanotechnol-10-930-s001.pdf]

BEILSTEIN JOURNAL OF NANOTECHNOLOGY

Supporting Information

for

In situ AFM visualization of Li–O_2 battery discharge products during redox cycling in an atmospherically controlled sample cell

Kumar Virwani, Younes Ansari, Khanh Nguyen, Francisco José Alía Moreno-Ortiz, Jangwoo Kim, Maxwell J. Giammona, Ho-Cheol Kim and Young-Hye La

Beilstein J. Nanotechnol. **2019,** *10,* 930–940. doi:10.3762/bjnano.10.94

Additional figure, table and movie captions

License and Terms: This is a supporting information file under the terms of the Creative Commons Attribution License (http://creativecommons.org/licenses/by/4.0). Please note that the reuse, redistribution and reproduction in particular requires that the authors and source are credited.
The license is subject to the *Beilstein Journal of Nanotechnology* terms and conditions: (https://www.beilstein-journals.org/bjnano)

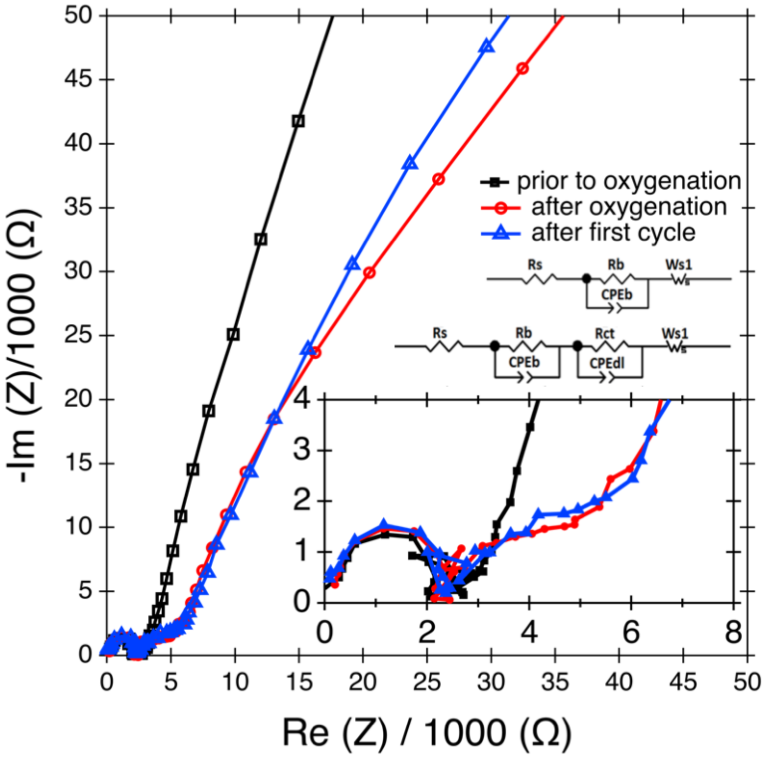

Figure S1: Electrochemical impedance spectroscopy curves collected before (square points) solvent oxygenation, after solvent oxygenation for two hours (circle points) and after the first discharge recharge cycle (triangle points). The EIS data collected for cell with 1 M $LiNO_3$ in TEGDME with ≈4600 ppm of water in the electrolyte. Fitting parameters used in the equivalent circuit mode shown in the inset are presented in Table S1. Prior to oxygenation equivalent circuit with constant phase element (CPEb) was used, rest of the EIS spectra were fit with equivalent circuits that included both CPEb and CPEdl.

Table S1: presents the EIS fitting parameters for the cell prior to oxygenation, after oxygenation, and after the first discharge and recharge cycle. As it can be inferred from the table, the Rs value after the first cycle is increased. This is attributed to an increase resistance in the electrolyte during cycling which perhaps is due to the reaction of the electrolyte with the discharge product. As shown in the table, the constant phase element (CPE) is defined by two values, CPE-T and CPE-P. The CPE is a simple distributed element which produces an impedance having a constant phase angle in the complex plane and the exponent P (in CPE-P) that determines this angle. A CPE-P value close to unity is a behavior similar to that of a capacitor. CPE-T is the capacitive values of CPE. The CPE-T value of the

cell after oxygenation is increased which is attributed to the changes in the passivating layer after oxygenation.

Water Content	Cell State	Rs/Ω	Rb/Ω	CPE-T/F	CPEb-P/F	Rct/Ω	CPEdl-T/F	CPEdl-P/F	W-R/Ω	W-P/Ω
**<20 ppm**	**no O_2**	119.9	1464	8.13×10^{-11}	1.125				7.3×10^{5}	0.407
**<20 ppm**	**O_2 added**	191.9	1404	3.18×10^{-11}	1.192				5.16×10^{5}	0.389
**<20 ppm**	**First cycle**	218.6	1307	2.03×10^{-11}	1.227				4.9×10^{5}	0.392
**2500 ppm**	**no O_2**	190.4	2310	1.87×0^{-10}	1.058	–	–	–	5.91×0^{5}	0.78
**2500 ppm**	**O_2 added**	186.8	2288	9.35×0^{-11}	1.103	2541	1.51×0^{-6}	0.878	2.54×0^{5}	1.35
**2500 ppm**	**First cycle**	248.1	2035	2.45×0^{-11}	1.205	3687	5.26×0^{-6}	0.678	3.33×0^{5}	1.00
**4600 ppm**	**no O_2**	220.9	1901	5.15×10^{-11}	1.153				8.45×10^{5}	0.367
**4600 ppm**	**O_2 added**	178.9	2220	3.18×10^{-11}	1.12	2043	8.69×10^{-7}	0.982	5.57×10^{5}	0.366
**4600 ppm**	**First cycle**	139.1	2007	2.82×10^{-11}	1.19	1888	2.3×10^{-6}	0.917	5.79×10^{-11}	0.370

Movie captions

Movie 1 (Supporting Information File 2)

AFM topography evolution on cathode surface during discharge and recharge in 1 M LiNO3 TEGDME and 5 µA discharge current (<20 ppm water): 54 time domain correlated electrochemical discharge (black, left Y axis) and recharge (blue, right Y axis) curves with topography images from simultaneous AFM scans. The 3 µm × 3 µm AFM images were collected in 1 M $LiNO_3$ in TEGDME with <20 ppm of water in the electrolyte at nine spiral spots. The background image is a 20 µm scan of the surface after the discharge charge cycle.

The discharge current was 5 µA. Nine movie frames prior to the start of the discharge depict the initial topography of glassy carbon. The red box denotes current electrochemical state and the corresponding AFM image. Lighter shade of red denotes the state 131 seconds prior to the current state. Pink shade denotes the state of the system 262 seconds prior to the current state. Electrochemical deposits appear to nucleate, grow and then eventually shrink on the glassy carbon surface as the cell goes through discharge and recharge.

Movie 2 (Supporting Information File 3)

AFM topography evolution on cathode surface during discharge and recharge in 1 M $LiNO_3$ TEGDME and 5 µA discharge current (≈2500 ppm water): 184 time domain correlated electrochemical discharge (black, left Y axis) and recharge (blue, right Y axis) curves with topography images from simultaneous AFM scans. The 3 µm × 3 µm AFM images were collected in 1 M $LiNO_3$ in TEGDME with ≈2500 ppm of water in the electrolyte at nine spiral spots. The background image is a 20 µm scan of the surface after the discharge charge cycle. The discharge current was 5 µA. Eight movie frames prior to the start of the discharge depict the initial topography of glassy carbon. Authors hypothesize that during EIS also the nanostructures form in the solution that over time settle on the glassy carbon. The red box denotes current electrochemical state and the corresponding AFM image. Lighter shade of red denotes the state 131 seconds prior to the current state. Pink shade denotes the state of the system 262 seconds prior to the current state. Nanostructured electrochemical deposits abruptly appear (≈4% capacity) on the cathode surface during the initial stages of discharge that appears to continue for another ≈1 µAh discharge. This point on the discharge curve is denoted by grey color rectangle in frames 17 and greater. Beyond that discharge capacity, smaller nanostructures appear to nucleate and grow on the cathode surface. The size of the deposits continues to increase along with fresh nucleation and growth on all areas until the cell discharge capacity of 11.6 µAh. During recharge the nanostructures abruptly disappear between 14% and 18% of the recharge capacity. The first point on the electrochemical recharge curve where the abrupt disappearance occurs is denoted by a grey rectangle. Beyond 18% recharge the surface of glassy carbon was free of deposits suggesting that the discharge products were suspended/dissolved in the electrolyte solution. As expected at the end of the charge the polishing marks on the glassy carbon surface line-up across the 3 µm and 20 µm scans.

Movie 3 (Supporting Information File 4)

AFM topography evolution on cathode surface during discharge and recharge in 1 M $LiNO_3$ TEGDME and 5 µA discharge current (≈4600 ppm water): 231 time domain correlated electrochemical discharge (black, left Y axis) and recharge (blue, right Y axis) curves with topography images from simultaneous AFM scans. The 3 µm × 3 µm AFM images were collected in 1 M $LiNO_3$ in TEGDME with ≈4600 ppm of water in the electrolyte at nine spiral spots. The background image is a 20 µm scan of the surface after the discharge charge cycle. The discharge current was 5 µA. Eight movie frames prior to the start of the discharge depict the initial topography of glassy carbon. Authors hypothesize that during EIS also the nanostructures form in the solution that over time settle on the glassy carbon surface. For higher water concentration the size is larger. The red box denotes current electrochemical state and the corresponding AFM image. Lighter shade of red denotes the state 131 seconds prior to the current state. Pink shade denotes the state of the system 262 seconds prior to the current state. Nanostructured electrochemical deposits abruptly appear (≈9 % capacity) on the cathode surface during the initial stages of discharge that appears to continue for another ≈1 µAh discharge. This point on the discharge curve is denoted by grey color rectangle in frames 26 and greater. Beyond that discharge capacity, smaller nanostructures appear to nucleate and grow on the cathode surface. The size of the deposits continues to increase along with fresh nucleation and growth on all areas until the cell discharge capacity of 24 µAh. During recharge the nanostructures abruptly disappear between 20% and 40% of the recharge capacity. The first point on the electrochemical recharge curve where the abrupt disappearance occurs is denoted by a grey rectangle. Beyond 40% recharge the surface of glassy carbon was free of deposits suggesting that the discharge products were suspended/dissolved in the electrolyte solution. As expected at the end of the charge the polishing marks on the glassy carbon surface line-up across the 3 µm and 20 µm scans.

Movie 4 (Supporting Information File 5)

AFM topography evolution on cathode surface during discharge only in 1 M $LiNO_3$ TEGDME and 350 nA discharge current (≈4600 ppm water): Subset of 316 out of 3167 time domain correlated electrochemical discharge (black, left Y axis) curves with topography images from simultaneous AFM scans. The 3 µm × 3 µm AFM images were collected in 1 M $LiNO_3$ in

TEGDME with ≈4600 ppm of water in the electrolyte at nine spiral spots. The background image is a 22 µm scan of the surface after the discharge. The discharge current was 350 nA. The red box denotes current electrochemical state and the corresponding AFM image. Lighter shade of red denotes the state 1180 seconds prior to the current state. Pink shade denotes the state of the system 2360 seconds prior to the current state. Most of the electrochemical deposits grow conformal to the glassy carbon surface as would be expected at such low discharge currents. Correspondingly the cell discharge capacity was measured to be ≈47 µAh. The movie represents 7 days of continuous data collection. This experiment helped establish the stability of the technique over multiple days of experimentation. This also helped quantify that tapping mode-scanning changes the topography only about 1.5 nm in 344 scans! As expected the surface of the glassy carbon is covered with conformal deposits after the discharge reaction.
